# Modeling and Experimental Research on Resistance Spot Welded Joints for Dual-Phase Steel

**DOI:** 10.3390/ma12071108

**Published:** 2019-04-03

**Authors:** Dawei Zhao, Yuanxun Wang, Peng Zhang, Dongjie Liang

**Affiliations:** 1 School of Mechanical and Vehicle Engineering, Linyi University, Linyi 276005, China; 2 Department of Mechanics, Huazhong University of Science and Technology, Wuhan 430074, China; wooshin@163.com (Y.W.); ldj_hust@126.com (P.Z.); 3 Guangxi Zhuang Autonomous Region Institute of Metrology & Test, Nanning 530007, China; dj75037882@163.com

**Keywords:** advanced high-strength steel, resistance spot welding, simulation analysis, nugget diameter

## Abstract

Dual-phase steel has been employed in the automotive industry as it has the advantages of high strength, satisfying ductility, low yield ratio, and so on. A novel framework for the weld nugget size prediction and control using finite element modeling and experimental research was proposed in this paper. The two-dimensional axisymmetric numerical analysis model was established and the phase transition on thermal expansion coefficient was taken into account. The whole welding process was simulated and discussed using thermal elastic-plastic theory. To validate the predictive methods of developed weld nugget size and confirmation experiments were implemented with the same input parameters in the ranges of process parameters. The simulated weld nugget sizes were in good agreement with the experimental results except for extreme welding conditions. The microstructure of the welding zone was also investigated based on metallographic experiments and temperature field analysis. The welding parameters were adjusted using the model proposed in this paper so as to obtain the nugget size with pull out failure mode.

## 1. Introduction

Currently, a great deal of attention has been focused on the automotive industry to reduce fuel consumption and gas emission by means of reducing the weight of vehicles. One such initiative is the development of welding joints with high-strength steels. As one kind of high-strength steel, dual-phase steels (DP500-1000) possess the advantages of high strength, good corrosion resistance, satisfying press performance, and so on, and have been gradually employed in modern car body manufacturing [1,2]. Resistance spot welding (RSW) is the main joining technique widely employed in car body manufacturing. It is a multifaceted process involving the metallurgy, mechanics, thermoelectricity, fluid-structure interaction, and many other coupling factors [3,4]. In recent years, a great amount of research work on the resistance spot welded joints of DP steel was available. Hayat et al. [5] evaluated the fracture toughness of galvanized DP600 steel sheets under RSW against the welding current and welding time with the thickness of 1.20 mm. It was observed from the results that the relationship between fracture toughness and welding current, welding time was a second-degree polynomial; fracture toughness of spot welds was not only dependent on the nugget diameter but also relied on sheet thickness, tensile rupture force, welding time, and current. Kaščák et al. [6] investigated the influence of the primary welding parameters on the weld quality by testing microhardness and tensile shear load bearing capacity of resistance spot welds of DP600 steel sheets. Huin et al. [7] characterized the fracture mechanisms for the RSW joints of two common steel grades DP600 and martensitic steel Usibor 1500. Cross tension and tensile shear tests were carried out, while the resulting strengths, microstructures, failure modes, and fracture mechanisms were compared. It was found that the failure load of heterogeneous spot welds increased with the nugget size and the sheet thickness regardless of the failure mode, as long as no splash defects occurred. Aktas et al. [8] studied the effect of welding current and welding time on mechanical properties of DP600 steel sheet joints to detect the optimum welding parameters for maximum joint strength. Kim et al. [9] used a conically shaped hollow electrode for the lower electrode to improve resistance spot weldability of three-sheet welding, including high strength steel. Shear strength test, analysis of welding signal, and weld cross-section were conducted. It was found that the proposed lower electrode had some advantages in welding the three-sheet welding compared with conventional electrodes or hollow electrodes. Hernández et al. [10] assessed the influence of polarity during resistance spot weld of dissimilar lap joints between DP 600 dual-phase steel and AISI 304 stainless steel on mechanical properties and failure modes. Jaber et al. [11] investigated the microstructure, failure mode transition, peak load, and energy absorption of spot welded DP600 dual-phase steel during the tensile-shear test. It was found that the welding current has profound effect on the load-displacement characteristics. 

In fact, the weld joints are accomplished by the effects of a series of welding process parameters, which mainly includes the welding current, electrode force, welding time, and so on [12,13,14]. The mechanical property of the weld assembly is basically dependent on the integrality of each welding joints. During the welding process, deformation of the workpieces, the stress and strain in the welding zone will be produced and changed. These mechanical properties have great influences on the performances of welding joints, including nugget formation and its failure strength [15]. Consequently, to understand the temperature distribution in the welding process, the deformation and phase transformation of the microstructure is extremely important. Nevertheless, the process of spot welding is such a highly non-linear, multi-parametered, and complicated process containing a large number of random factors. Only through experimental study can we obtain insightful and knowledgeable information about welding process [16]. Combining the finite element numerical simulation with experimental research provides a very valuable method for welding process parameters recommendation to get sound welding joints in actual production. 

The main objective of this study is to develop a two-dimensional axisymmetrical thermal-electrical finite element model to simulate the welding process for DP600 steel sheets using the finite element software Ansys (Canonsburg, PA, USA). The simulation results were compared with the test results to verify the validity of the model. The microstructure of the welding zone was also investigated based on metallographic experiments and temperature field analysis of the thermal-electrical-mechanical finite element model. The appropriate welding parameter combinations guaranteeing the welding quality were finally proposed. The numerical simulation can predict the nugget diameter with the changes of process parameters in the welding process rather than based on experiences without theory, which also reduces the expensive experimental cost and provides guidance for further research in the actual production.

## 2. Materials and Methods

The material investigated was 1.7 mm thick ferrite-martensitic dual phase steel DP600. The typical chemical composition of DP600 steel (wt%) is C 0.11, Mn 1.6, Si 0.182, Ni 0.027, Nb 0.0037, Ti 0.002, Cr 0.34, Mo 0.098, and V 0.0035. The basic mechanical properties of DP600 steel [17] are shown in Table 1. The welding samples for the experiments were made by shearing form the steel sheet plates against the direction of rolling; the dimensions were 40 × 120 mm and with overlapping 40 mm. Then the samples were cleaned by acetone and the welding tests were performed using pneumatic spot welding machine (Panasonic Welding Systems Company, Tangshan, China). Two-pulse alternating welding current was employed to produce the welded joints; the frequency of alternating current was 50 Hz. The copper alloyed truncated electrode was used and its diameter was 6 mm. The sizes of steel sheets for lap welding were 120 mm × 40 mm and the overlapped length was 40 mm. The welding process parameters were determined on the basis of literature review and preliminary welding tests [18]. The holding time and squeeze time were kept at 10 cycles (1 cycle = 0.02 s), the welding process parameters employed in the welding experiments were listed in Table 2. The electronic universal testing machine was used to conduct quasi-static tensile shear tests for the welding joints. The loading speed was 10 mm/s. The mechanical properties of the specimens were obtained according to the load-displacement curves. Peak load is the maximum value of load-displacement curve, while the maximum displacement is its corresponding displacement value. The total failure energy is the area under load-displacement curve [19], while the penetration rate can be obtained according to Zhao et al. [20]. The failure modes of samples were determined from the rupture interfaces of the samples after the tensile shear tests. Metallographic cross section for each weld was polished by automatic grinding and polishing machine. Abrasive cloths with different size of particles were used for the polishing. After polishing, they were etched using natal for nugget size measurement and microstructure observation to judge the welding quality. The Axiovert 200 Microscope produced by Carl Zeiss (Heidenheim, German) was used for metallographic observations.

During the finite element analysis process, the electrode force was first applied to the steel sheets and it resulted in initial deformations and contact area. Then the temperature distribution was considered for an increment from fully coupled electrical thermal finite element analysis. The electrical and thermal boundary conditions were also considered in the model. The Joule heating resulted from the welding current at the faying surfaces of DP600 steel sheets and electrode were calculated. Consequently, the temperature distributions in the welding zone would be achieved and the final results were obtained using the mechanical analysis after several loops. Therefore, the heat flow problem of resistance spot welding is a heat conduction related to melting and subsequent solidification. Although the nature of the heat flow is three-dimensional, it can also be simulated as the form of the two-dimensional axisymmetric model, because the circular section of the electrode can be applied on both current and compressive force at the same time. The advantage of the axisymmetric formula is that its symmetry *θ*, the azimuth angle, allows for heat transfer and flow changes in radial (*r*) and vertical (*z*) directions [21]. Therefore, in current work, thermal analysis is considered to be an axisymmetric transient heat conduction.

Supposing the temperature is constant, the control equation of transient heat conduction in cylindrical coordinate system can be expressed as [22]:

(1) $$\frac{\partial }{\partial r}\left(\gamma \cdot \frac{\partial T}{\partial r}\right)+\frac{\gamma }{r}\cdot \frac{\partial T}{\partial r}+\frac{\partial }{\partial z}\left(\gamma \cdot \frac{\partial T}{\partial z}\right)+q_{v}=C_{p}\cdot \rho \frac{\partial T}{\partial t}$$

Among them, *z* is the axial coordinate of the cylindrical coordinate system, and *r* is the radial coordinate. *q_v_*, *t*, *T*, *γ*, *ρ* and *C_p_* respectively indicate heat yield, time, temperature, thermal conductivity, density, and specific heat, whose units are all international units, and all of them are functions of temperature.

The steady-state current conduction equation in the sheet electrode system can be obtained by in the aspect of electrical voltage [21]:

(2) $$\frac{\partial }{\partial r}\left(\frac{1}{\rho_{E}}\cdot \frac{\partial U}{\partial r}\right)+\frac{1}{\rho_{E}\cdot r}\cdot \frac{\partial U}{\partial r}+\frac{\partial }{\partial r}\left(\frac{1}{\rho_{E}}\cdot \frac{\partial U}{\partial z}\right)=0$$

where $\rho_{E}$ is the resistivity of the material, *U* is the electrical potential.

**Figure 1:**
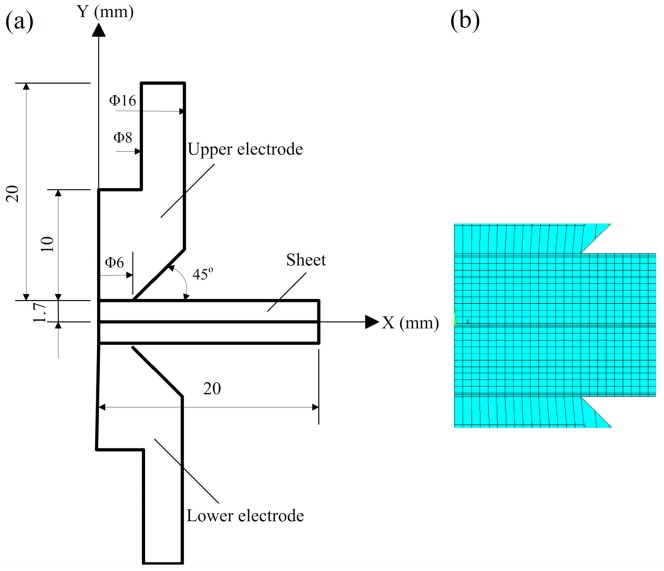
Finite element model employed to simulate RSW process: (**a**) overall view; and (**b**) enlarged view.

A two-dimensional axisymmetric model was established as the thermoelectrical analysis geometry model to analyze the welding process using the software Ansys, as shown in Figure 1. The radius of the model was 20 mm and the thickness of model was 3.4 mm, which was two times of the DP600 sheets thickness. Thermoelectric coupling elements were employed to mesh the model and solid elements were utilized to simulate the thermo-elastic-plastic behavior of the faying surfaces between the sheets and electrodes. The thickness of the solid element at the contact of the sheet-sheet surface was 0.035 mm and the one at the sheet-electrode surface was 0.07 mm. 

The solid element Plane42 was selected in the squeeze stage. The contact surfaces between the electrode/workpiece and workpiece/workpiece with the action of the electrode force were flexible contacts, and the deformation and movement of the material were tracked by the elements TARGE169/CONTA172. The thermoelectric element Plane67 was selected for the electrical-thermal coupled analysis. The contact resistance is represented by a physical unit attached onto the surfaces of workpieces and electrodes by artificially adding a layer of 1/20 of the workpiece thickness. The structural element Plane42 was selected in the thermal analysis stage. As the same in the squeeze phase, the elements TARGE169/CONTA172 were employed to monitor the deformation and movement of the contact surface.

**Figure 2:**
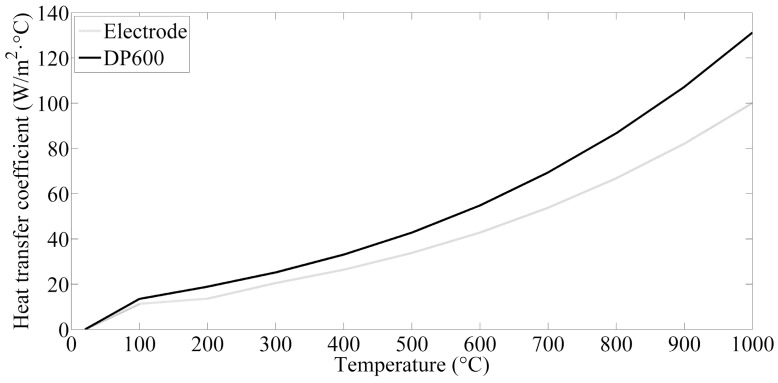
Surface coefficients of heat transfer of electrodes and steel.

The cooling water temperature inside the electrodes was assumed to be 10 °C and the ambient temperature was 20 °C. As the temperature was above 1500 °C, DP600 sheet began to melt and this critical value was thought to be the threshold temperature for nugget formation. The surfaces of the electrode and thin sheets pass heat to the air by convection. The convective heat transfer coefficient for the cool water in the electrodes is 4.187 × 10^4^ W/m^2^·K. The surface heat dissipation coefficients for electrode and metal sheets are presented in Figure 2.

**Figure 3:**
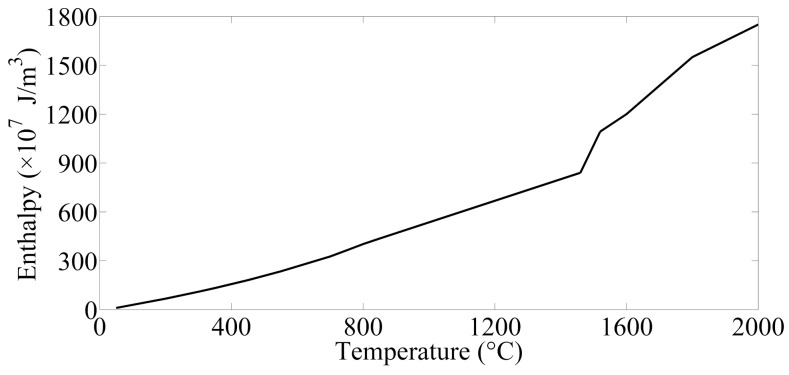
Enthalpy values of steel sheets with different temperatures.

Additionally, one of the most remarkable characteristic of the welding process is that the coexistence of solid and liquid phase, solid state phase transition and solid-liquid phase transition in the welding zone, which is supposed to produce an effect on the temperature field. Consequently, enthalpy was employed to solve the problem of latent heat during the thermoelectric analysis and modeling the welding process [21]. Enthalpy is defined as:

(3) H=∫ρC(T)dT

where *ρ* is the density of welding joints, *C*(*T*) is the specific heat of weldment. According to the definition of definite integral, Equation (4) can be written as follows:

(4) $$H\left(T_{n}\right)=\rho \overset{n}{\underset{i=1}{\sum }}\frac{C_{i}+C_{i+1}}{2}\Delta T_{i}$$

where *n* is the number of iterations, *T_n_* is the final temperature, *H*(*T_n_*) is the enthalpy as the corresponding temperature is *T_n_*, C*_i_* is the initial specific heat at the *i_th_* interval, *C_i_*_+1_ is the final specific heat at the *i_th_* interval, Δ*T_i_* is the interval of temperature. In this case, the enthalpy of steel sheets with different temperatures can be solved and it is presented in Figure 3.

**Figure 4:**
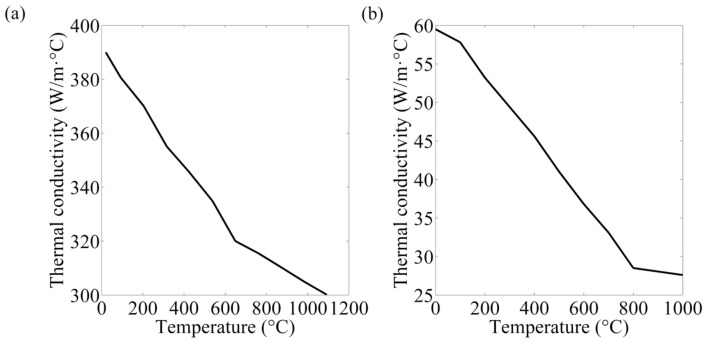
Thermal conductivity of the electrode (**a**) and DP600 (**b**).

**Figure 5:**
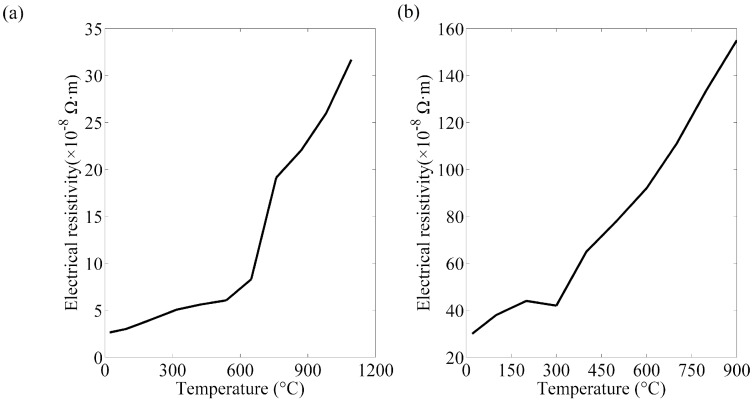
Resistivity of the electrode (**a**) and DP600 (**b**).

**Figure 6:**
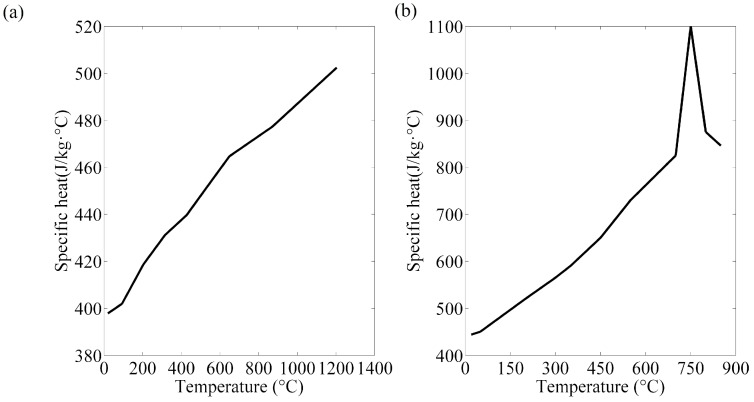
Specific heat of the electrode (**a**) and DP600 (**b**).

**Figure 7:**
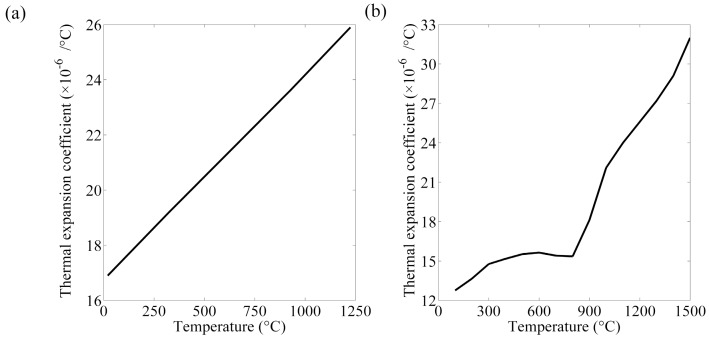
Thermal expansion coefficient of the electrode (**a**) and DP600 (**b**).

The thermal, mechanical, and electrical properties of the base material and electrode had to be available so as to obtain accurate results [23]. As the electrical and physical values are affected by the temperature, they are commonly acquired from literature review and supposed to be homogeneous. Mechanical properties of the electrode and steel sheets at room temperature were listed in Table 3 [24,25]. The thermo-electrical properties parameters of the steels and electrode are presented in Figure 4, Figure 5, Figure 6 and Figure 7 [26,27,28]. The properties of DP600 with very high temperature were employed according to Bézi et al. [29].

**Figure 8:**
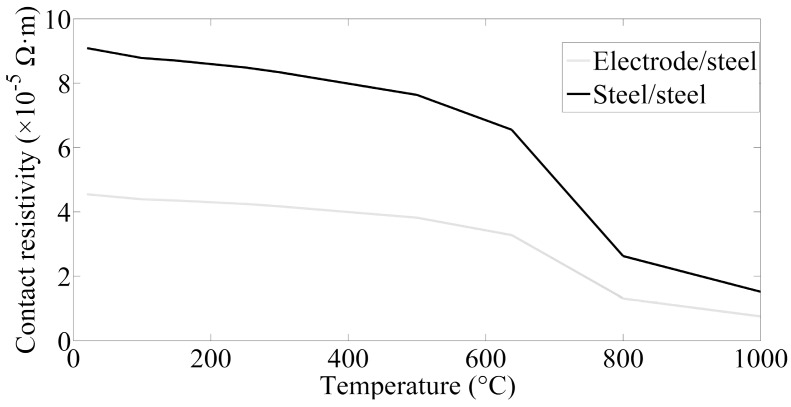
Contact resistivity (F = 3.5 kN).

The contact resistance is another factor affecting the Joule heat in the welding process and it is normally obtained through measuring in experiment. The initial value of contact resistance at room temperature can be worked out via Equation (5) [30]. In most cases, as for a specific welding condition, the electrode force is set as a constant value, the contact resistance can be simplified to the function of temperature, as shown in Equation (6) [31,32]. It can be translated into the contact resistivity by means of Equation (7):

(5) $$R_{c}=rF^{-m}$$

(6) $$R_{T}=\frac{T_{m}-T}{T_{m}-T_{0}}\cdot R_{c}$$

(7) $$\bar{\omega }=\frac{R_{T}\cdot S}{L}$$

where *r* is the coefficient indicating the nature and the surface state of DP600 steel sheets, it is generally acknowledged the value is 1.375 × 10^−2^ between copper and steel (electrode and steel contact surface), and 2.75 × 10^−2^ between steels (workpiece interfaces) at room temperature [30]. *R_c_* is the initial contact resistance at room temperature, *m* is an index, the value of which is frequently in the range of 0.5 to 1, the value of *m* is set as 0.7 in this paper. *F* is the electrode force, *T_m_* is melting temperature of DP600 steel and its value can be set as 1500 °C, *T*_0_ is room temperature which set as 20 °C, *R_T_* is contact resistance as the temperature is *T*, *S* is cross-sectional area of welding current path, *L* is the length of contact area, *ω* is the resistivity. Hence, the reference contact resistivity values at various temperatures can be determined by linear interpolation [33] and the specific contact resistances of interfaces of sheet/sheet and electrode/sheet are shown in Figure 8.

**Figure 9:**
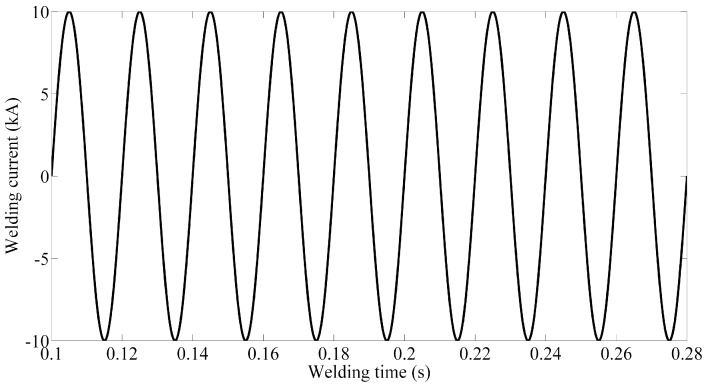
The waveform of welding current.

The main process parameters determining the quality of spot welding are electrode pressure, welding current and welding time. As the simple sinusoidal alternating current was employed in the welding experiments and the frequency is 50 Hz. During the cooling stage in the welding process, the welding current was cut off. Thus, the welding current in the finite element analysis can be expressed as follows (shown in Figure 9):

(8) $$I=\{\begin{array}{c} I_{m}sin100\pi t,0\leq t\leq t_{m}\\ 0,t>t_{m} \end{array}$$

## 3. Results

**Figure 10:**
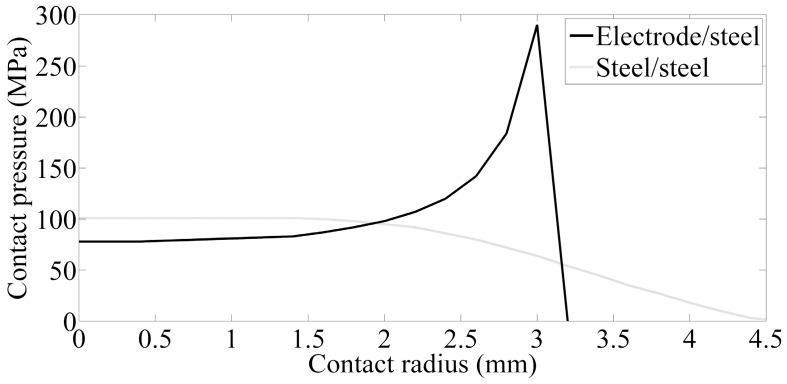
Contact pressure distribution of the welding joints in the squeeze stage.

Figure 10 shows the contact pressure of the electrode/sheet and sheet/sheet contact surfaces as the electrode force is 3.5 kN in the squeeze stage. The holding time is set as 10 cycles. The pressure stress is unevenly distributed along the contact radius, which is caused by its uneven axial stress distribution. As for the electrode force and DP600 steel sheet, the contact pressure distributes more smoothly near the center of electrodes, and then it increases rapidly until stress concentration is observed on edge of the electrodes. The contact pressure rapidly reduces to zero once it is beyond the electrode edge. The conductive area of the contact point between the electrode and steel sheet is the same as the electrode face area. The pressure stress of the contact surface between the upper steel and lower steel slowly declines until it reaches zero, the virtual contact radius, which is about 4.6 mm. It is much larger than the electrode radius.

**Figure 11:**
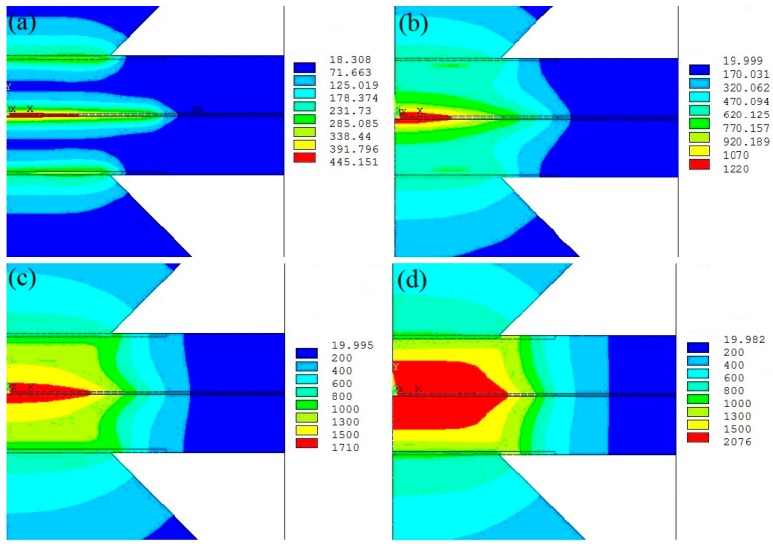
Temperature profile of the welding zone in the welding process (**a**) 0.007 s, (**b**) 0.05 s, (**c**) 0.13 s, and (**d**) 0.28 s.

Figure 11 shows the temperature distributions in different welding time calculated in the welding process. The corresponding welding current is 10 kA, the electrode force is 3.5 kN, andthe welding time is 14 cycles (0.28 s). From Figure 11a, at the initial stage, the temperature at the surfaces of sheet/sheet and electrode/sheet is much higher than other places, the contact resistance is the main factor generated welding heat and its value is the largest at the interfaces of sheet/sheet (Figure 8). The highest temperature at this stage is about 450 °C at this time. As the carbon content of DP600 steel is about 0.11%, the temperature of austenitic initialization *A_c_*_1_ is 717 °C, and the temperature of complete transition into austenitic *A_c_*_3_ is 854 °C [33,34]. The microstructure of the welding joint at this stage is the same as DP600 steel since its temperate was below *A_c_*_1_. When the welding time is 0.05 s, the temperature at the interfaces of electrode/sheet is much smaller than that of sheet/sheet. From now on, the dominant factor of the influence on welding heat is the bulk resistance of the upper and lower sheets, and the electrical resistivity of DP600 steel is much larger than copper alloy electrode. At this stage, the largest temperature at the faying surfaces is about 1220 °C and it is lower than the melting point 1500 °C, while the two sheets are in plastic adhesion state with the action of the electrode pressure imposing on them. Then, as time goes on and the application of welding current continues, the temperature at the faying surfaces of the welding zone is above 1500 °C, molten metal appears and gradually enlarges until the welding current is cut off. The temperature of most of the welding zone is above *A_c_*_3_ and the austenitic is filled with this region. The temperature of the electrode surface is smaller than 500 °C and, meanwhile, the shape of molten metal is oval, which is caused by the cooling water in the electrodes. The heat affected zone is usually identified as the zone of austenitic initialization contained. The region covered by the contact radius of the electrode and DP600 sheet is the heat affected zone.

**Figure 12:**
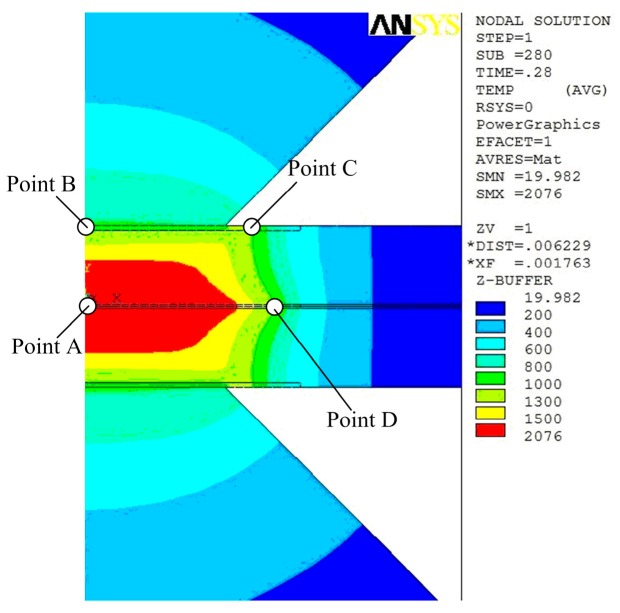
Four key points selected.

**Figure 13:**
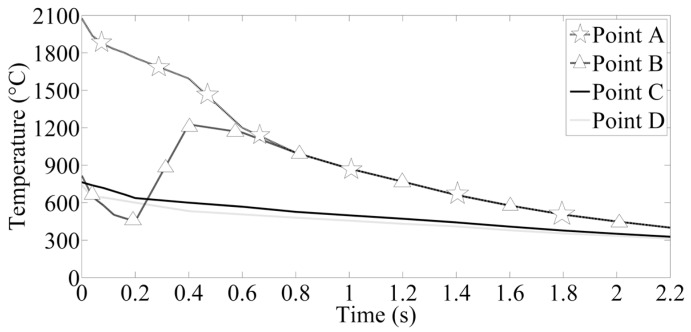
Temperature profile of the four points after the welding current was cut off.

Figure 12 shows the key point selected in the welding zone in order to investigate the cooling rate of the welding joints in the cooling stage after the welding current is cut off. Figure 13 presents the changes of the temperature on the four points. As the welding current is cut off, the electrode force is still imposed on the welding specimen with the purpose of relaxation of residual stress; meanwhile the cooling water is employed in order to cool the welding zone. This stage has an unignorable effect on the microstructure transformation of the fusion zone. At the initial stage, the cooling rate is very high and the molten area progressively grows smaller. At the final stage, it is much smaller than before but the maximum temperature is still very high. At the beginning, the minimum temperature of the four points is about 740 °C and it is above *A_c_*_1_, and the temperatures of points B, C, and D are all below *A_c_*_3_, which means these areas are not completely austenitized while the area near point A is completely austenitized. In the first 0.2 s, the cooling rates of the four points are all higher than the critical cooling rate of austenitic complete transformation into martensite [35]. As the welding joints were removed, the temperature of point B increases rather than decreases and the paces of temperature changes are synchronized with point A without the effect of cooling water.

Table 4 lists the nugget size predicted by finite element modeling and measured. As the welding peak current is respectively 6 kA, 8 kA, and 10 kA, the results of simulation analysis can reflect the heat affected zone (HAZ) of the welding joints, the simulated nugget size is consistent with the actual results, with the relative error about 2%. However, as the welding current is extremely high (12 kA), the simulated nugget size is larger than the observed value and the relative error is about 10%. As extremely large welding current is employed in welding process, the molten metal is prone to splash from the welding zone and the nugget size should be diminished due to expulsion. However, this factor can’t be considered in the thermoelectricity transient analysis of the welding process.

**Figure 14:**
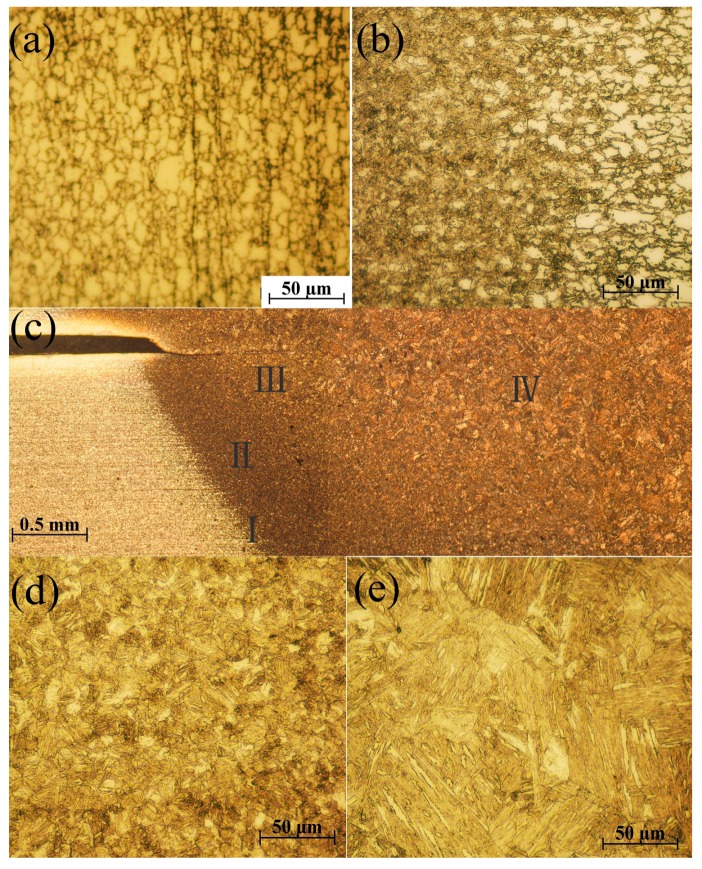
The microstructure of the welding joints: (**a**) dual-phase region (I in Figure 14c); (**b**) fine grained region (IIin Figure 14c); (**c**) metallographic structure of the weld nugget; (**d**) overheated zone (III in Figure 14c); and (**e**) fusion zone (IV in Figure 14c).

**Figure 15:**
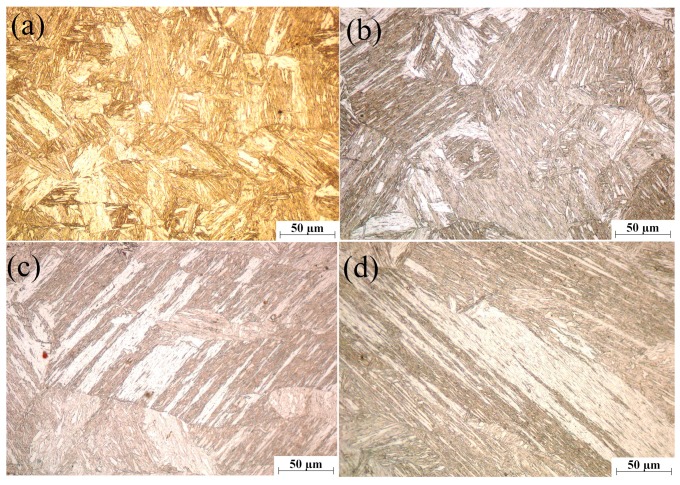
The microstructures of welding joints with different dwell periods above *Ac1*: (**a**) 156 ms; (**b**) 234 ms; (**c**) 256 ms; and (**d**) 267 ms.

Figure 14 presents the microstructure of the welding joints. The fusion zone and heat affected zone are heated above *A_c1_* under the influence of heat generated by resistance and welding current, while ferrite begins to change into austenite. In the cooling stage, the austenite transforms into martensite, which has been discussed above. The microstructures of fusion zone are mainly composed of martensite structure, as shown in Figure 14e. The cooling rates of the whole welding joints are all larger than the critical cooling rate of hypoeutectoid steel martensitic phase transformation. When it cools to a temperature lower than *M_s_*, non-invasive martensitic phase transformation will be developed in the region. The carbon content of DP600 steel is less than 0.2%, and the result of martensitic transformation is that the fusion zone is filled with big lath martensite as it cools to room temperature. The heat affected zone can be divided into the overheated zone (Figure 14d), fine grained region (Figure 14b), and dual-phase region (Figure 14a) outward along the fusion zone. The overheated zone is uniformly distributed with lath martensite, which is completely in agreement with the fusion zone. Fine grained region is generally composed of fine and uniform martensite, and this region is farther from the center of nugget and the microstructure is almost filled with austenitic with the action of welding current heating. But the dwell time above the austenitizing temperature time is shorter, the grain size is not fully grown, the region is mainly composed of slight lath martensite, a small amount of ferrite and retained austenite after cooling. The microstructure of base metal does not change in the whole welding process; it mainly consists of fine ferrite and martensite. Ferrite is soft phase, controlling the material formability, while martensite is hard phase, in charge of the hardness and strength of the material. Figure 15 exhibits the microstructures of four fusion zones with different welding parameters. As the dwell time above austenitic initialization *Ac*1 is respectively 156 ms, 234 ms, 256 ms, and 267 ms according to finite element modeling. The size of the martensite grain increases successively.

**Figure 16:**
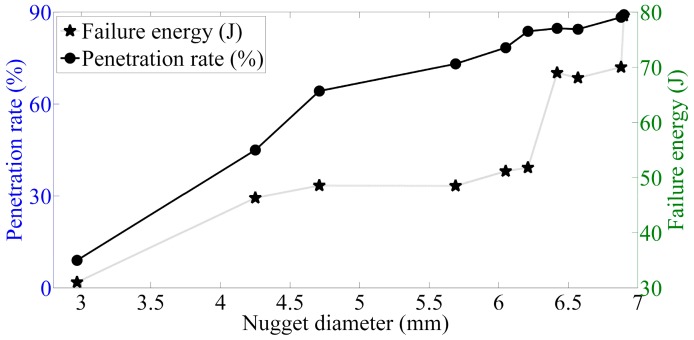
Relationships between the nugget diameter, penetration rate, and failure energy.

**Figure 17:**
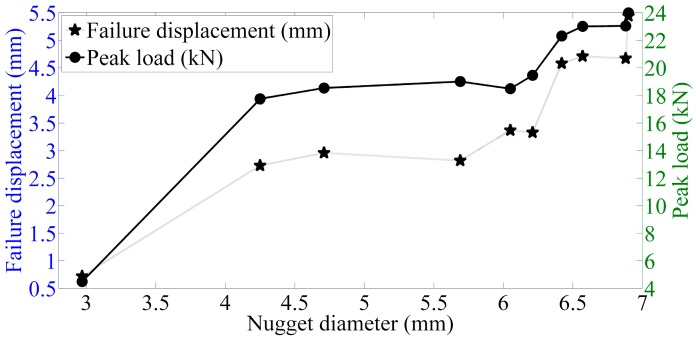
The relationships between the nugget diameter, peak load, and failure displacement.

The main quality characteristics of welding joints consist of nugget size, heat affected zone width and height, peak load, indentation depth, and failure modes in the tensile-shear test. On one hand, it can be seen from Figure 16 and Figure 17 that the maximum displacement, peak load, penetration rate and failure energy are monotonically increasing with the fusion size. On the other hand, the influences of welding parameters on the nugget size are consistent with the influences of welding parameters on mechanical properties of joints. Then it logically follows that the welding parameters directly affect the nugget size, while the mechanical properties of the sample are directly controlled by the fusion size. Table 5 lists the Pearson correlation coefficients between the nugget size and penetration rate, peak load, failure energy and failure displacement, which were calculated using the software Matlab 2013a. Thus, in a comprehensive view, the most direct control factor of the mechanical properties of the resistance spot welding joints is the nugget size. 

Based on the experimental results, the effects of nugget size on the mechanical properties of resistance spot welding are emphatically analyzed. As Figure 16 and Figure 17 present the relationships between the nugget size and the peak load, maximum displacement, and failure energy, the welding joints can be divided into three categories according to the nugget diameter: cold weld, undersized weld and good weld. A cold weld is usually generated as the welding heat in the welding zone is so little that the steel just starts to melt, even though the temperature has not reached the melting point. In this case, the mechanical performances of this kind weld are very poor. As for the undersized weld, the steel plates thermally fused with each other; however, as it is still unsatisfying, as it cannot bear relatively large forces. As for undersized welds, a brittle fracture is the main form of efficiency loss. The sufficient heat input leads the adjacent surfaces to melt, resulting in an increasing nugget volume. The larger the nugget size is, the greater the maximum displacement, peak load, and the failure energy will be. The maximum displacement, peak load, and failure energy increase significantly with the nugget diameter as the welding joints belong to undersized weld or good weld; while in the undersized weld region, the mechanical properties increased slowly. This shows that changing the nugget size has a significant effect on the mechanical properties of the welding joints. During the loading failure process, as the nugget size gradually increases, the peak load is obviously increasing, and shear deformation also increases significantly.

It is well documented that the size of the fusion zone is the key physical weld attribute controlling the mechanical properties of spot welds among all the welding quality indices [36]. The failure modes of the welding joints include: the pullout failure (PF) mode and interfacial failure (IF) mode. The PF mode is preferred as it indicates a higher-fracture energy and better mechanical property. The larger the nugget size is; the better mechanical properties of the welding joints will be improved and the PF mode will occur. As the nugget belongs to a cold weld, its failure mode is completely IF. As the nugget gets larger, its failure mode transfers from the IF mode into the PF mode. However, the costs of the weld assembly, for example, the welding heat input demands, will be also increased in the meantime. The critical nugget size *D* induced the IF transforming into the PF mode is considered to be just satisfying without increasing the productive cost (*t* is the thickness of the steel sheets) [18]:

(9) D=3.51t

To accomplish the goal of ensuring the welding quality, while reducing welding costs as much as possible, it is necessary to optimize the welding process parameters through controlling the weld nugget size. At present, setting and determining the welding process parameters are commonly based on lots of experiments and work experience. 

**Figure 18:**
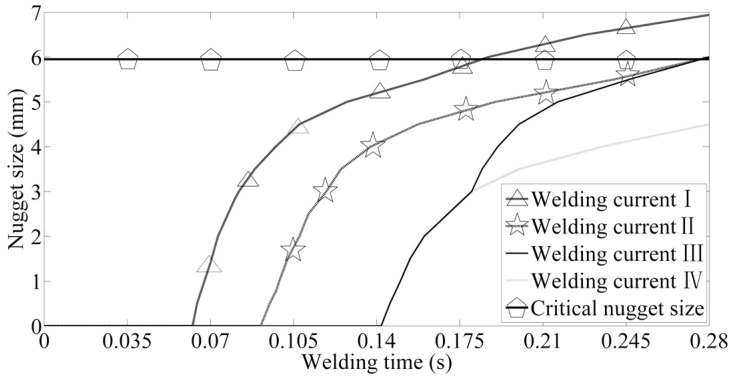
Controlling the nugget size.

If the welding parameters are given, the nugget size of the welding joints can be predicted by finite element analysis. For example, the thickness of DP600 steel sheets is 1.7 mm, the welding time is 14 cycles, the welding current is 8 kA, and the electrode force is 3.5 kN. The nugget size is 4.4 mm, which is much smaller than the critical nugget size 5.97 mm. It indicates that, this welding parameters combination cannot guarantee the welding quality. It is necessary to adjust the weld nugget size through changing the welding process parameters. Based on the finite element analysis, there are several methods controlling the nugget diameter as following. The welding current can be adjusted with the purpose of increasing the nugget size close to 6.0 mm in order to guarantee the welding quality. The welding current can be changed into 9 kA, and the final nugget size is about 6.0 mm (Figure 18 curve II). Improving the welding current peak to 10 kA in later period (nine cycles) to change the rangeability of weld nugget in the slow-growth stage is another method (shown as curve III in Figure 18). Curve I is the nugget diameter growth in the welding process as the welding current is 10 kA, the final nugget size is 6.94 mm, and it is satisfactory. The peak load of the welding joints whose nugget size is slightly smaller the critical nugget is far less than that of critical nugget, even if there is a little difference between the their nugget sizes. However, when the nuggets diameters are above the critical value, the mechanical performances of the welding joints with very different nuggets are close to each other [37]. From this point of view, nugget size (curve II and curve III in Figure 18) slightly larger than the critical nugget value not only indicates relatively sound mechanical performances, but also saves energy and boosts efficiency. The costs of Curve I seem to be a bit high.

## 4. Conclusions

(1) The contact radius of the electrode/sheet contact surfaces is basically equal to the electrode radius in the preloading stages, while the value of sheet/sheet contact surface is much larger than that.

(2) The formation of the nugget in theoretical analysis undergoes three stages: plasticity adhesion, rapid growth, and slow growth. 

(3) Close correlation is observed between simulated thermal fields and the microstructure changes of joints. The longer the dwell time temperature is larger than the austenitic initialization *A_c1_* of the joints, the coarser the lath martensite that will be generated.

(4) It should be pointed out that the error is about 10% as the welding current is very large and the expulsion occurs. When the expulsion occurs, molten metal splashes from the welding zone and the nugget size is smaller than expected, however, this can’t be considered in the finite element modeling.

(5) The accuracy of the physical experiments and numerical simulations is within the acceptable range. It is a real potential for the simulated results to be adopted by a wide range of users for process planning in modern manufacturing. 

## Figures and Tables

**Table 1:** Mechanical properties of DP600 steel.

Tensile Strength	Specified Non-Proportional Elongation Stress	Percentage Elongation after Fracture	Plastic Strain Ratio	Strain Hardening Exponent
*R_m_*	*R_p_*_0.2_	*A*80	*r*	*n*
MPa	MPa	%	%	%
633	407	23	67	14

**Table 2:** Welding parameters employed during the RSW process.

Welding Parameters	Symbol	Unit	Value
Electrode force	*F*	kN	2.0, 2.5, 3.5, 4.0
Welding time	*T*	cycles	8, 11, 14, 17
Welding current	*I*	kA	6, 8, 10, 12

**Table 3:** Mechanical properties of electrode and DP600 at room temperature.

Materials	Yield Strength	Elasticity Modulus	Strain Hardening Modulus	Poisson’s Ratio	Density
Symbol	*σ_s_* (MPa)	*E* (GPa)	*E_t_* (MPa)	*ν*	*ρ* (kg/m^3^)
Electrode	230	115	566	0.35	8900
DP600	360	213	611	0.3	7800

**Table 4:** Comparison of simulated results and experimental results.

Welding Time	Welding Current	Electrode Force	Measured Nugget Size	Simulated Nugget Size	Relative Error	Maximum Temperature at the Nugget Center
*T*	*I*	*F*	*D*	*D*_1_	Δ	*T_max_*
cycles	kA	kN	mm	mm	%	°C
11	10	2.0	4.71	5.10	8.28	1765
		2.5	5.69	5.80	1.93	1867
		3.5	6.42	6.20	3.43	1952
		4.0	6.21	6.40	3.06	2000
14	6	3.5	-	-	-	1302
	8		4.25	4.40	3.53	1770
	10		6.88	6.94	0.87	2076
	12		6.90	7.60	10.14	2286
8	10		6.05	5.50	9.09	1800
17			6.57	6.80	3.50	2185

**Table 5:** Correlation coefficients of the five welding quality indicators.

Correlation Coefficient	Nugget Diameter	Penetration Rate	Peak Load	Failure Energy	Failure Displacement
Nugget diameter	1.00	0.97	0.90	0.88	0.92

## References

[1] Wang X., Liu B., Liu W., Zhong X., Jiang Y., Liu H. (2017). Investigation on the mechanism and failure mode of laser transmission spot welding using PMMA material for the automotive industry. Materials.

[2] Fazaeli A., Ekrami A., Kokabi A.H. (2016). Microstructure and mechanical properties of dual phase steels, with different martensite morphology, produced during TLP bonding of a low C-Mn steel. Met. Mater. Int..

[3] Thakur A.G., Nandedkar V.M. (2014). Optimization of the resistance spot welding process of galvanized steel sheet using the Taguchi method. Arab. J. Sci. Eng..

[4] Amirthalingam M., van der Aa E.M., Kwakernaak C., Hermans M.J.M., Richardson I.M. (2015). Elemental segregation during resistance spot welding of boron containing advanced high strength steels. Weld. World.

[5] Hayat F., Sevim I. (2012). The effect of welding parameters on fracture toughness of resistance spot-welded galvanized DP600 automotive steel sheets. Int. J. Adv. Manuf. Technol..

[6] Kaščák L., Spišák E., Gajdoš I. (2015). Influence of welding parameters on the quality of resistance spot welded joints of DP600 steels. Key Eng. Mater..

[7] Huin T., Dancette S., Fabrègue D., Dupuy T. (2016). Investigation of the failure of advanced high strength steels heterogeneous spot welds. Metals.

[8] Aktas S., Ozsarac U., Aslanlar S. (2012). Effect of spot welding parameters on tensile properties of DP600 steel sheet joints. Mater. Manuf. Process..

[9] Kim D., Yu J., Rhee S. (2016). Effect of a conically shaped hollow electrode on advanced high strength steel in three-sheet resistance spot welding. Int. J. Precis. Eng. Manuf..

[10] Espinel Hernández A., Sánchez Roca A., CarvajalFals H., AntoniFerraresi V., Oliveira Vilarinho L. (2016). Influence of polarity on mechanical properties of dissimilar resistance spot welds of DP 600/AISI 304 steels. Sci. Technol. Weld. Join..

[11] Jaber H.L., Pouranvari M., Salim R.K., Hashim F.A., Marashi S.P.H. (2017). Peak load and energy absorption of DP600 advanced steel resistance spot welds. Ironmak. Steelmak..

[12] Podržaj P., Simončič S. (2014). A machine vision-based electrode displacement measurement. Weld. World.

[13] Podržaj P., Jerman B., Simončič S. (2016). Poor fit-up condition in resistance spot welding. J. Mater. Process. Technol..

[14] Mvola B., Kah P., Martikainen J., Suoranta R. (2016). Dissimilar high-strength steels: Fusion welded joints, mismatches, and challenges. Rev. Adv. Mater. Sci..

[15] Zhang X., Yao F., Ren Z., Yu H. (2018). Effect of welding current on weld formation, microstructure, and mechanical properties in resistance spot welding of CR590T/340Y galvanized dual phase steel. Materials.

[16] Moghanizadeh A. (2016). Evaluation of the physical properties of spot welding using ultrasonic testing. Int. J. Adv. Manuf. Technol..

[17] Kaščák Ľ., Kubík R. (2014). Influence of welding parameters on the properties of spot welded joints of dual-phase steels. Transf. Inovácií.

[18] Zhao D., Wang Y., Liang D., Zhang P. (2016). Modeling and process analysis of resistance spot welded DP600 joints based on regression analysis. Mater. Des..

[19] Rao S.S., Chhibber R., Arora K.S., Shome M. (2017). Resistance spot welding of galvannealed high strength interstitial free steel. J. Mater. Process. Technol..

[20] Zhao D., Wang Y., Sheng S., Lin Z. (2014). Multi-objective optimal design of small scale resistance spot welding process with principal component analysis and response surface methodology. J. Intell. Manuf..

[21] Jagadeesha T., Jothi T.S. (2017). Studies on the influence of process parameters on the AISI 316L resistance spot-welded specimens. Int. J. Adv. Manuf. Technol..

[22] Mirzaei F., Ghorbani H., Kolahan F. (2017). Numerical modeling and optimization of joint strength in resistance spot welding of galvanized steel sheets. Int. J. Adv. Manuf. Technol..

[23] Mikno Z., Bartnik Z. (2016). Heating of electrodes during spot resistance welding in FEM calculations. Arch. Civ. Mech. Eng..

[24] Chrysochoos A., Berthel B., Latourte F., Pagano S., Wattrisse B., Weber B. (2008). Local energy approach to steel fatigue. Strain.

[25] Zhao Z., Yao L., Guo H. (1993). Copper and Copper Alloys Handbook.

[26] Tsai C., Dai W., Dickinson D. (1991). Analysis and development of a real-time control methodology in resistance spot welding. SAE Trans..

[27] Baumer R., Adonyi Y. (2009). Transient high-frequency welding simulations of dual-phase steels. Weld. J..

[28] Schenk T., Richardson I.M., Kraska M., Ohnimus S. (2009). Non-isothermal thermomechanical metallurgical model and its application to welding simulations. Sci. Technol. Weld. Join..

[29] Bézi Z., Baptiszta B., Szávai S. (2014). Experimental and numerical analysis of resistance spot welded joints on DP600 sheets. Weld. Mater. Test..

[30] Gould J.E. (1994). Modeling primary dendrite arm spacings in resistance spot welds, part I-modeling studies. Weld. J..

[31] Gould J.E. (1994). Modeling primary dendrite arm spacings in resistance spot welds, part II-experimental studies. Weld. J..

[32] Pakkanen J., Vallant R., Kičin M. (2016). Experimental investigation and numerical simulation of resistance spot welding for residual stress evaluation of DP1000 steel. Weld. World.

[33] Ramazani A., Bruehl S., Abbasi M., Bleck W., Prahl U. (2016). The effect of bake-hardening parameters on the mechanical properties of dual-phase steels. Steel Res. Int..

[34] Hayat F. (2011). Comparing properties of adhesive bonding, resistance spot welding, and adhesive weld bonding of coated and uncoated DP 600 steel. J. Iron Steel Res. Int..

[35] Ramazani A., Mukherjee K., Abdurakhmanov A., Abbasi M., Prahl U. (2015). Characterization of microstructure and mechanical properties of resistance spot welded DP600 steel. Metals.

[36] Wang X.J., Zhou J.H., Yan H.C., Pang C.K. (2018). Quality monitoring of spot welding with advanced signal processing and data-driven techniques. Trans. Inst. Meas. Control.

[37] Tamizi M., Pouranvari M., Movahedi M. (2017). Welding metallurgy of martensitic advanced high strength steels during resistance spot welding. Sci. Technol. Weld. Join..

